# The Economics of Animal Health: A 25-Year Bibliometric Analysis

**DOI:** 10.3390/ani15203006

**Published:** 2025-10-16

**Authors:** Arzu Peker, Şükrü Orkan, Luisa Magrin, Severino Segato

**Affiliations:** 1 Department of Animal Health Economics and Management, Faculty of Veterinary Medicine, Ankara University, Ankara 06110, Türkiye; agokdai@ankara.edu.tr (A.P.); orkan@ankara.edu.tr (Ş.O.); 2 Department of Animal Medicine, Production and Health, University of Padova, 35020 Legnaro, Italy; severino.segato@unipd.it

**Keywords:** animal health economics, bibliometric analysis, cost-effectiveness, economic burden, livestock diseases

## Abstract

**Simple Summary:**

Animal diseases not only cause suffering among animals but also very often result in huge economic problems for farmers and society. They can reduce productivity, restrict trade, and impose extra costs on governments and consumers. Understanding these economic outcomes is crucial for creating effective policies and enhancing global animal health. In this paper, we have examined the evolution of research in the economics of animal health over the last twenty-five years. To accomplish this, we examined over a thousand scientific papers to create a map of the primary contributors, the issues they concentrate on, and the ways researchers cooperate across countries. We found that most studies have focused on wealthy countries, yet countries most burdened by animal diseases may have limited research conducted. Our results suggest that more balanced research, closer international collaboration, and the development of new tools, such as advanced models, can make economic studies of animal health more valuable for decision-makers, producers, and society.

**Abstract:**

Economic implications of livestock diseases extend far beyond direct treatment costs and affect productivity, trade, and public health. Despite the growing recognition of animal health economics, a comprehensive analysis of its research landscape has been lacking. Therefore, this study employs bibliometric techniques to systematically analyze research on the economics of animal health between 2000 and 2024 using data extracted from the Web of Science Core Collection. A total of 1070 peer-reviewed publications were analyzed to map publication trends, influential authors, research themes, and international collaborations. The results showed that after 2014, the research output increased steadily to a peak in 2018, thus illustrating the increased global interest in economic evaluations of livestock diseases. The USA, UK, and the Netherlands emerged as key contributors, whereas low-income regions showed low research output, indicating an equity gap for animal health economics studies. The most frequently used keywords were “economics”, “cost–benefit analysis”, “economic impact”, “foot-and-mouth disease”, and “vaccination”, with increasing focus on zoonotic diseases. Coauthorship network analysis demonstrated that the institutions are well connected in Europe and North America, but research from developing countries has remained mostly fragmented. However, notable research gaps were discovered: advanced modelling approaches were underutilized, and the translation of economic research into policy was limited. This work highlights the increasing interdisciplinary nature of animal health economics, while emphasizing the need for broader species coverage, stronger international collaboration, and deeper methodological innovation. These insights provide a foundation for guiding future research priorities and shaping evidence-based policies in animal health economics.

## 1. Introduction

The intersection of animal health and economics has gained increasing significance in understanding and addressing global problems related to animal health, food security, and economic sustainability. Animal disease can impose important financial constraints on livestock productivity, trade, and public health [1]. Animal health economics aims to place a value on these effects through cost–benefit analysis, economic modelling, and policy assessment, thereby assisting decision-makers in developing efficient disease control interventions [2]. With antimicrobial resistance, zoonotic disease, and climate change transforming the livestock sector, understanding the economic effects of animal health measures has never been so critical [3].

The economic costs of animal disease extend beyond direct expenses, such as treatment costs and mortality-associated loss of income, to include indirect expenses, including trade limitations, reduced productivity, and sector-wide impacts on agricultural markets [4]. Especially for major transboundary and zoonotic diseases, devastating costs have been imposed on governments and stakeholders all over the world [1]. In addition, novel zoonotic infections continue to pose risks to the world’s economies, further warranting the application of combined economic studies that integrate epidemiological data and economic modelling methods [5]. As the veterinary profession has adopted more One Health paradigms, which focus on the interconnectedness of animal, human, and environmental health, economic analysis has become much more prominent in veterinary decision-making [6].

Previous bibliometric studies in related domains, such as agricultural economics and veterinary epidemiology, have provided useful insights into research trends; however, none have systematically examined the development of animal health economics itself. Bibliometric analysis, which employs quantitative methods to examine the scientific literature, offers a powerful means for tracking research advancements, collaborations, and future trends in animal health economics [7]. Previous bibliometric studies in related areas of agricultural economics and epidemiology have established those useful observations regarding the evolution of research needs over time can be gained, as well as areas that require investigation [8].

Therefore, this study aims to apply bibliometric techniques as a means of systematically mapping the state of research into the economics of animal health from 2000 to 2024. In particular, this study aims to identify trend issues in publication content, influential authors and institutions, research clusters, and international cooperation relations at the macro-level. In doing so, the paper synthesizes the literature on the subject, identifying some gaps, including the relative neglect of low-income regions, lessons for future research agendas, and approaches to evidence-informed policy-making in animal health economics.

## 2. Materials & Methods

This study employed a bibliometric approach to investigate animal health economics research from 2000 to 2024. The data were mined using the Core Collection of the Web of Science (WoS), a widely utilized and current scholarly database, to perform bibliometric analysis. To ensure a focused dataset, the following search string was applied in the Title/Abstract/Keywords (TS) field:

TS = (“economic impact” OR “economic burden” OR “cost analysis” OR “economic evaluation” OR “financial loss” OR “cost effectiveness” OR “economic analysis” OR “economic losses” OR “cost–benefit” OR “economic modeling” OR “decision analysis”) AND TS = (“animal health” OR “livestock diseases” OR “veterinary diseases” OR “zoonotic diseases” OR “PPR” OR “FMD” OR “brucellosis” OR “rabies” OR “avian influenza” OR “bovine tuberculosis” OR “mastitis” OR “lameness” OR “ketosis” OR “metritis” OR “leptospirosis” OR “BVD” OR “coccidiosis” OR “Pox” OR “scrapie” OR “animal welfare” OR “biosecurity” OR “compensation payments” OR “indemnity payments” OR “vaccination” OR “disease control” OR “disease eradication” OR “surveillance” OR “prevention program” OR “control program” OR “test and slaughter” OR “movement restriction”)

The search was conducted on 25 March 2025, ensuring coverage of all relevant articles published between 2000 and 2024. To refine the dataset, we applied the following inclusion and exclusion criteria:

### 2.1. Inclusion and Exclusion Criteria

To ensure the relevance and quality of the dataset, we included peer-reviewed journal articles and reviews published in English between 2000 and 2024, with a primary focus on the economic aspects of animal health. Opinion pieces, editorials, and non-research reports, as well as studies unrelated to animal health economics, were excluded. After screening and applying these criteria, a final dataset of 1070 publications was obtained for bibliometric analysis. Full details of the inclusion and exclusion criteria, as well as the screening parameters, are provided in the Supplementary Material (Table S1, Figure S1).

### 2.2. Validation

To validate the reliability of our search strategy, we conducted a parallel search in Scopus using the same query string, time range (2000–2024), document types (articles and reviews), and language filter (English) as applied in Web of Science (WoS). The search yielded 35,491 records in Scopus and 19,156 in WoS, reflecting differences in classification scope and indexing practices. After applying category filters and screening in WoS, 1070 relevant articles were identified. These were then cross-checked in Scopus using a fuzzy matching approach, revealing that 1035 were also indexed there, demonstrating strong thematic consistency across platforms. Although both databases are bibliometrically compatible, WoS was selected for in-depth analysis due to its more precise subject filtering and better integration with BibExcel software (Version 2016-02-20).

### 2.3. Descriptive Bibliometric Analysis

The software BibExcel, a bibliometric tool for processing and extracting publication data, was used to perform a bibliometric analysis of the downloaded articles. The analysis encompassed several indicators. The analysis covered several indicators. First, publication trends were examined by analysing the number of articles published annually between 2000 and 2024. Second, we analyzed the geographical distribution of research outputs by country. Author contributions and impact were assessed by identifying the most cited authors, measured by their h-index scores. A keyword analysis was conducted to identify the most common terms in the field. We also examined journals and publishers in terms of publication volume and citation ratios. Finally, institutional distribution was analyzed by considering the organizations with the highest number of contributing authors. The outcome of the descriptive analyses was tabulated and visualized in pie charts and line graphs using Excel.

### 2.4. Network Analysis and Visualization

To map the intellectual and collaborative structure of research in animal health economics, we used VOSviewer (Version 1.6.20), which is a widely used software for scientific network visualization. Several network analyses were performed to gain insights into the field. First, a coauthorship network was analyzed to identify the most influential researchers and examine institutional collaborations. Next, a co-occurrence analysis was conducted to map the connections among co-occurring keywords, revealing thematic clusters within the research domain. Lastly, a citation network analysis was conducted to investigate the citation relationships between the most prominent papers in the subject field, thereby shedding light on the academic influence and knowledge flow in animal health economics. In VOSviewer, the minimum restriction was set according to citation and co-occurrence frequency to enable the construction of an efficient structure for specific nodes.

## 3. Results

### 3.1. Descriptive Bibliometric Analysis

#### 3.1.1. Annual Growth in Publication Numbers (2000–2024)

**Figure 1:**
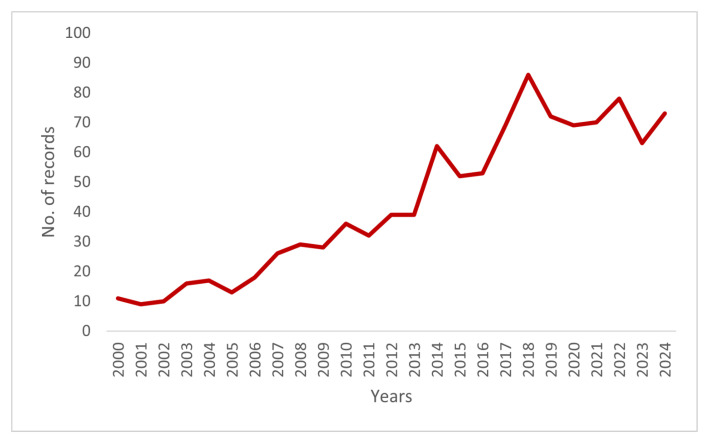
Number of publications over the years (2000–2024).

The line graph (Figure 1) shows an overall upward trend in publications between 2000 and 2024, with a notable increase after 2005 and a peak in 2018. Despite minor fluctuations, the overall trend indicates a steady rise in research output in animal health economics.

#### 3.1.2. Geographic Distribution of Articles

**Figure 2:**
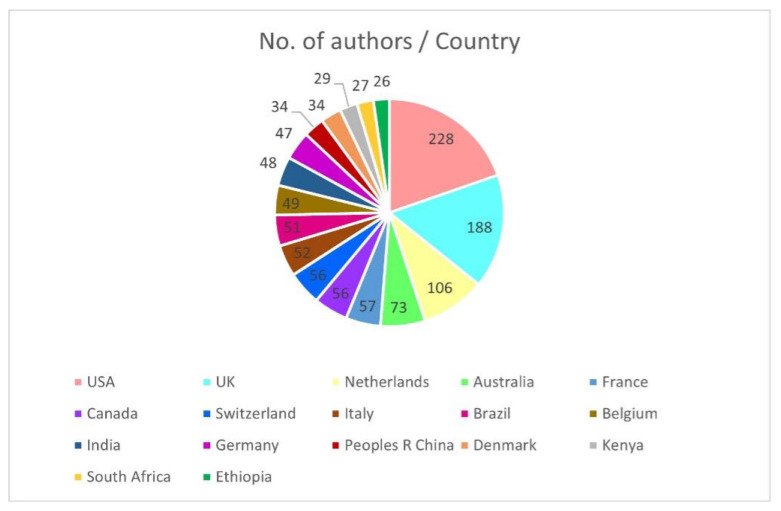
Country-wise research output.

Figure 2 gives the number of authors by country, indicating the share of research contributions. The first one belongs to the USA, with 228 authors, followed by the UK with 188 authors, and the Netherlands with 106 authors. The contributions from Australia are also significant, with 73 authors. Other countries, including France, Canada, Switzerland, Italy, and Brazil, achieve intermediate contributions, with between 50 and 57 authors. Countries such as India, Ethiopia and South Africa contribute to the field, although in small numbers of publications. Despite the variations, the chart depicts a mixed effort worldwide in research, indicating dominance in some leading countries.

#### 3.1.3. Author Contributions and Influence

**Figure 3:**
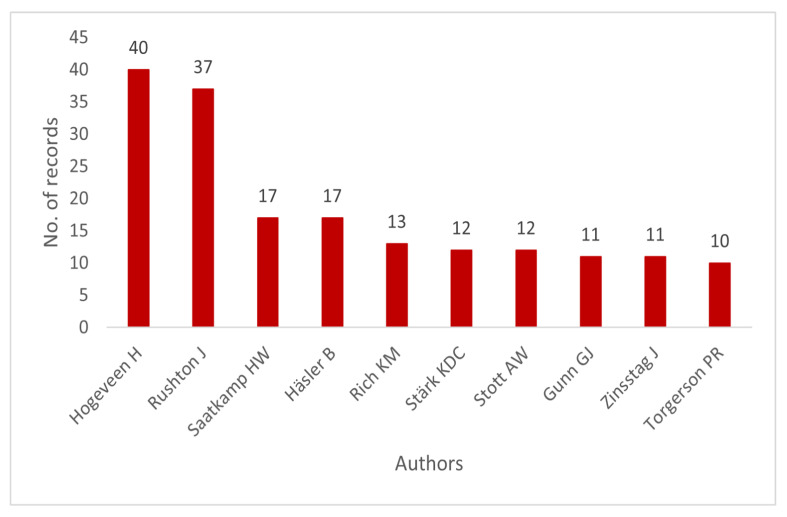
Top 10 most prolific authors.

The analysis of the most prolific authors in animal health economics is given in Figure 3. Based on the results, Hogeveen H dominates the field with 40 publications, followed by Rushton J with 37 publications. Other notable contributors include Saatkamp HW (17), Häsler B (17), Rich KM (13), Stärk KDC (12), Stott AW (12), Gunn GJ (11), Zinsstag J (11), and Torgerson PR (10).

Table 1 shows the top 10 authors with the highest h-index and their citation information. The h-index analysis indicates that Hogeveen H is the most prominent author in animal health economics, with an h-index of 22, 40 publications, and a total of 2667 citations. The next significant contributor is Rushton J, who follows with an h-index of 17, 37 publications and 1742 citations. Other noteworthy contributions came from Häsler B (h-index: 10), Rich KM (h-index: 10), and Stott AW (h-index: 9), all of whom continue to display importance in the discipline.

From the overall pattern, it follows that some researchers have a greater publication count than others, but their citation impact contradicts this. While Zinsstag J and Halasa T have fewer publications, they maintain strong citation records (1778 and 1025, respectively), which suggests that their studies have had a significant impact. These underscore the fact that flows of publications and citations are equally important in determining the weight of animal health economics.

The most cited references are those in animal health economics that emphasize foundation studies that have greatly influenced this field. The most-cited references are listed in Table 2. Rushton, J. (1999) [9] and McInerney, J. (1992) [10], with 50 citations, Knight-Jones, TJD. (2013) [3], with 44 citations, and Seegers, H. (2003) [11], with 42 citations, are the leading references.

#### 3.1.4. Keyword Analysis

**Figure 4:**
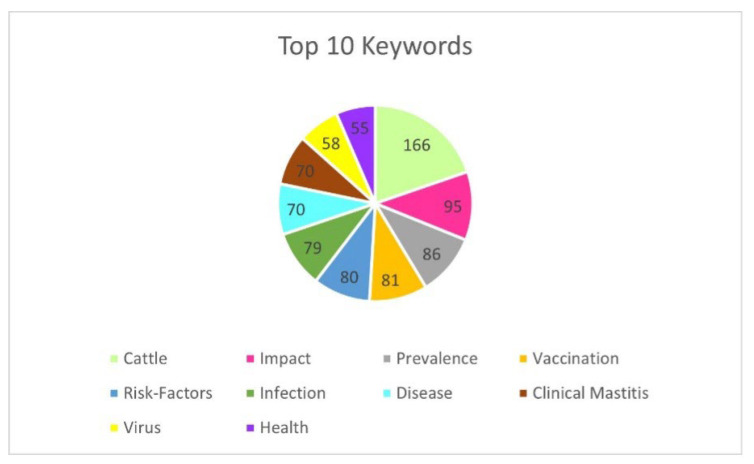
Top 10 most frequently used keywords.

Keyword analysis shows significant thematic issues in the field of veterinary science in relation to economics, illustrated in Figure 4. The most frequently used keywords are “cattle”, “impact”, “prevalence”, “vaccination”, and “risk-factors”, which probably indicate some interest in the cost and financial aspects of risk factors and disease control in animals, particularly in cattle.

#### 3.1.5. Journal and Publisher Analysis

**Figure 5:**
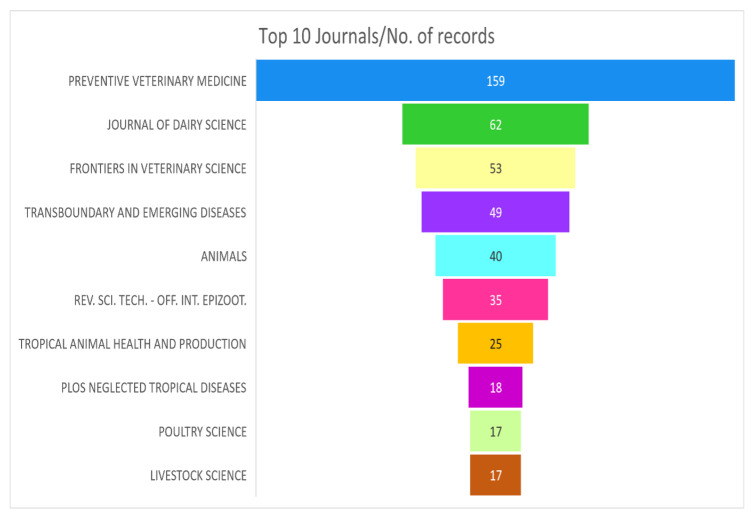
Top 10 journals with the highest number of publications.

Figure 5 shows the top 10 journals with the highest number of publications. *Preventive Veterinary Medicine* is the leading journal with 159 articles, followed by the *Journal of Dairy Science* with 62 articles. It has been observed that a high percentage of research in the field focuses on disease prevention and the financial implications for dairy production. Other notable journals include *Frontiers in Veterinary Science* (53), *Transboundary and Emerging Diseases* (49) and *Animals* (40).

The journals such as *Revue Scientifique et Technique-Office International Des Epizootıes* (REV.SCI.TECH.-OFF.INT.EPIZOOT) (35) and *Tropical Animal Health and Production* (25) show a growing relevance of animal health economics studies in developing regions and in tropical livestock production systems. In addition, *Plos Neglected Tropical Diseases* (18), *Poultry Science* (17) and *Livestock Science* (17) show a continued focus on production efficiency and economic viability.

**Figure 6:**
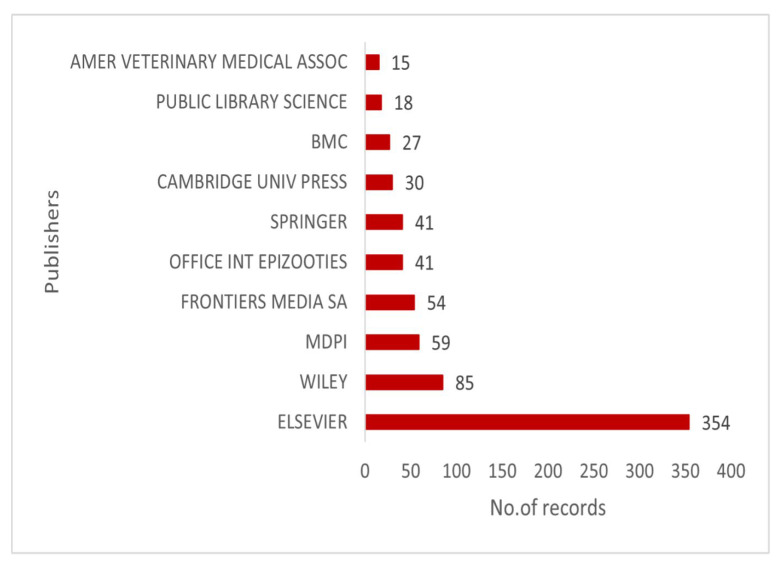
Top 10 publishers with the highest number of publications.

The top 10 publishers with the highest number of publications are presented in Figure 6. The major publisher, Elsevier, has reported 354 publications. Wiley and MDPI have contributed significantly to this field, with 85 and 59 articles, respectively. Other major publishers are Frontiers Media SA, Office Int Epizooties and Springer.

#### 3.1.6. Citation Impact and Distribution

**Figure 7:**
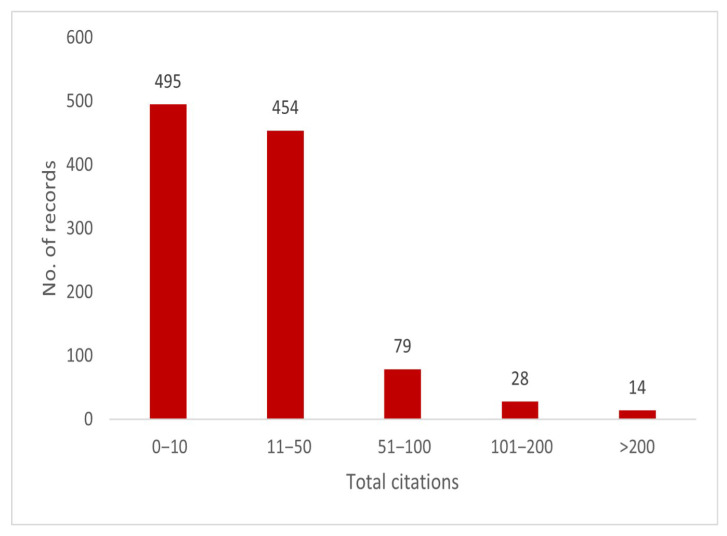
Distribution of articles based on citation ranges.

The distribution of citations in animal health economics research is given in Figure 7. Our results indicate that the majority of articles fall in the 0–10 citation range (495) and 11–50 citation range (454 items), implying that many published studies have relatively low to medium citation counts. By contrast, a smaller number of highly influential papers fall into the 51–100 citations category (79 articles), the 101–200 citations category (28 articles), and the >200 citations category (14 articles).

#### 3.1.7. Institutional Distribution

Wageningen University emerges at the top in analyzing institutional contributions to animal health economics, with 49 authors in total, thus indicating its concentration on animal health and agricultural economics. Utrecht University (35 authors) and the University of Liverpool (29 authors) also show higher profiles in their participation in the field.

**Figure 8:**
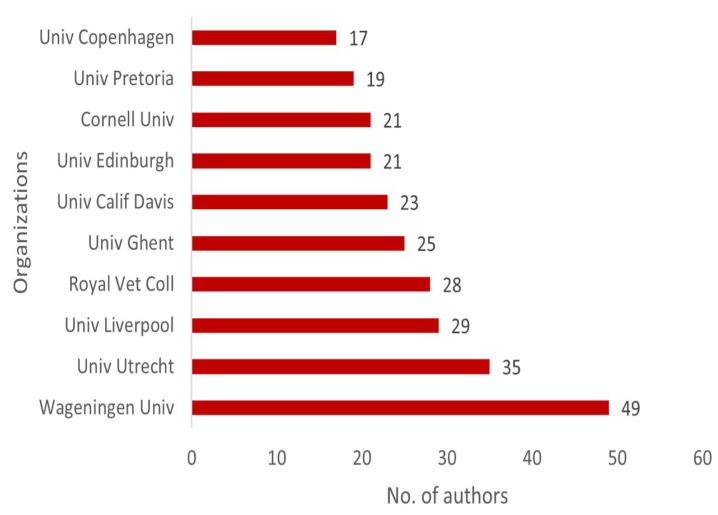
Top 10 organizations with the highest number of contributing authors.

Other noteworthy contributors are the Royal Veterinary College (28 authors), University of Ghent (25 authors), and the University of California, Davis (23 authors). They exemplify the role of European research institutes in stimulating economic evaluations of animal health. Institutions such as Cornell University and the University of Pretoria (each with 19–21 authors) also demonstrate the global nature of animal health economics, with equal representation from both developed and developing countries (Figure 8).

### 3.2. Network Analysis and Visualization

#### 3.2.1. Coauthorship Network: Authors

**Figure 9:**
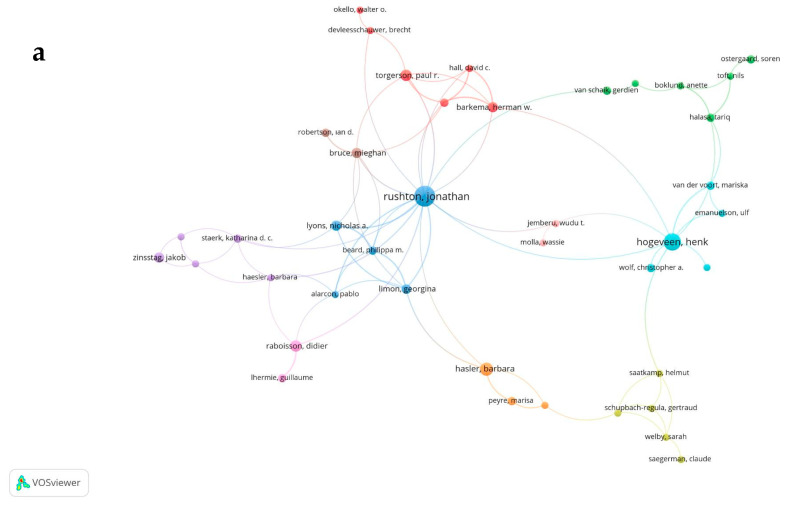

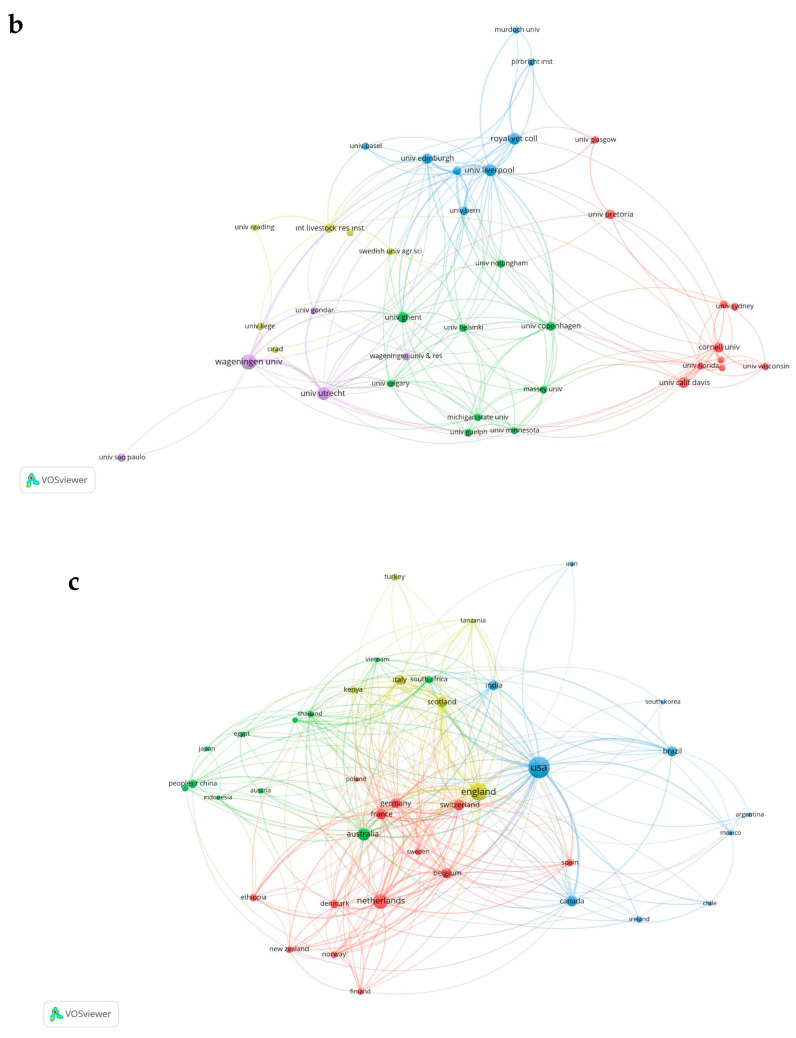
(**a**) Network visualization of coauthorship among authors. (**b**) Network visualization of coauthorship among organizations. (**c**) Network visualization of coauthorship among countries.

In the analysis of authors, the threshold has been settled at a minimum of 3 publications per author. Only 151 authors occurring in 10 clusters met the threshold. Rushton J emerges as a highly collaborative author with a total of 23 documents and a total link strength of 26, indicating robust partnerships within this network. However, Hogeveen H is another author with a high number and strength of links. His number of publications, links and total link strength are 16, 9, and 11, respectively (Figure 9a).

#### 3.2.2. Coauthorship Network: Organizations

In the analysis of organisations, the threshold has been established at a minimum of 10 publications per organisation. Only 38 organisations occurring in 5 clusters met the threshold. Institutional-level analysis highlights Utrecht University (Total Link Strength = TLS = 46), University of Liverpool (TLS = 39), Wageningen University (TLS = 38), and University of Edinburgh (TLS = 22) as major contributors, with strong inter-institutional links. European institutions, particularly in the Netherlands and the UK, show significant collaboration, while North American universities, including Cornell University and the University of Florida, form a separate but interconnected network (Figure 9b).

#### 3.2.3. Coauthorship Network: Countries

At the country level, the threshold has been settled for a minimum of 10 publications per country. Only 40 countries occurring in 4 clusters met the threshold. It has been determined that England (TLS = 210), the USA (TLS = 163) and Scotland (TLS = 102) were dominant countries in animal health economics, forming extensive international collaborations. Countries such as the Netherlands (TLS = 100), Switzerland (TLS = 91) and France (TLS = 84) also exhibit strong coauthorship (Figure 9c).

#### 3.2.4. Keyword Co-Occurrence: Research Themes

**Figure 10:**
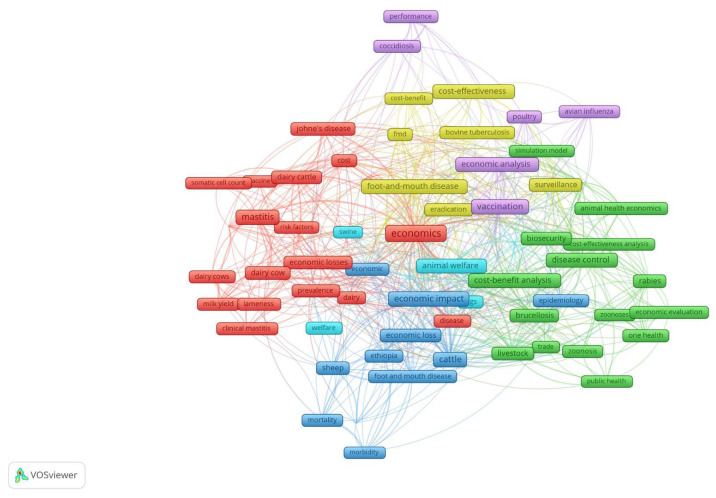
Keyword co-occurrence and research themes.

For the keyword co-occurrence analysis, the threshold for the minimum number of occurrences of a keyword has been settled to 10 occurrences. Only 70 keywords occurring in 6 clusters met the threshold (Figure 10). In cluster 1 (red color), the most used keyword was “economics” with 88 occurrences. In cluster 2 (green color), the most common keyword was “cost–benefit analysis” with 34 occurrences. In cluster 3 (blue color), the most used keyword was “economic impact” with 57 occurrences. In cluster 4 (yellow color), the most used keywords were “foot-and-mouth disease” with 35 occurrences. In cluster 5 (purple color), the most common keyword was “vaccination” with 47 occurrences. In cluster 6 (light blue color), the most used keyword was “animal welfare” with 40 occurrences.

#### 3.2.5. Keyword Co-Occurrence and Temporal Evolution

**Figure 11:**
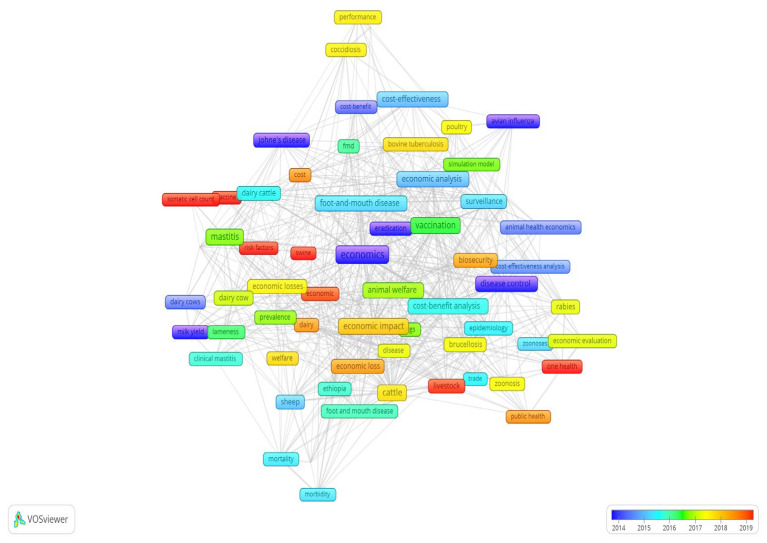
Keyword Co-Occurrence and Temporal Evolution.

An overlay visualization (Figure 11) was generated to display how research themes evolved over time. The color of each keyword represents the average publication year, from dark blue (earlier studies) to red (recent studies). Earlier research mainly addressed production-related losses such as mastitis, lameness, and milk yield, while later studies increasingly focused on cost–benefit analysis, foot-and-mouth disease, vaccination, animal welfare, and One Health. This temporal pattern reveals a clear shift from descriptive cost studies toward preventive, welfare-oriented, and interdisciplinary economic analyses in animal health.

#### 3.2.6. Citation Network: Journals

**Figure 12:**
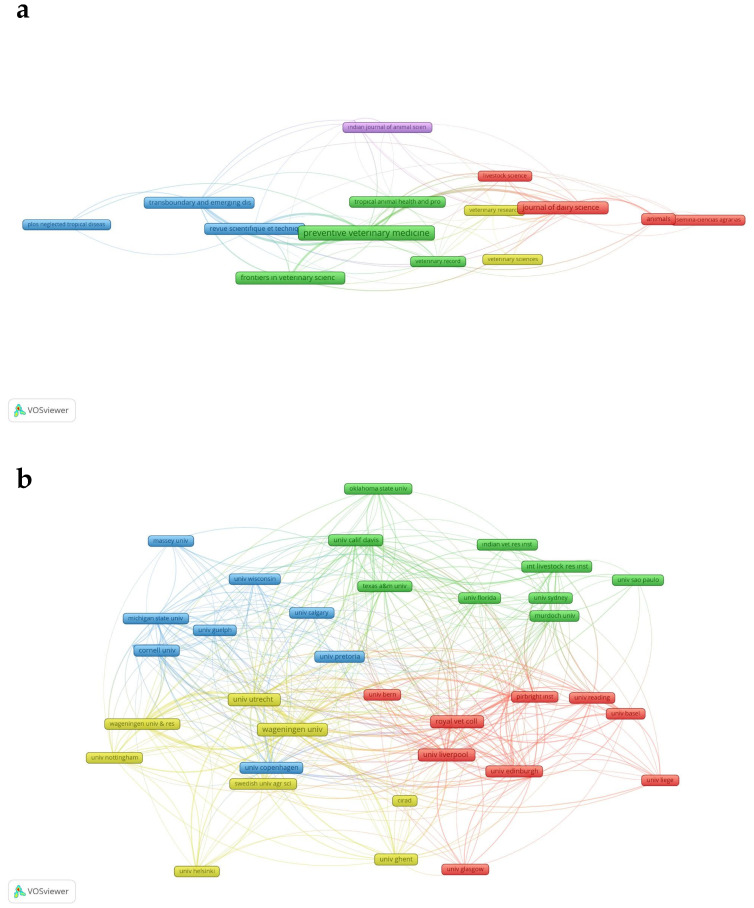

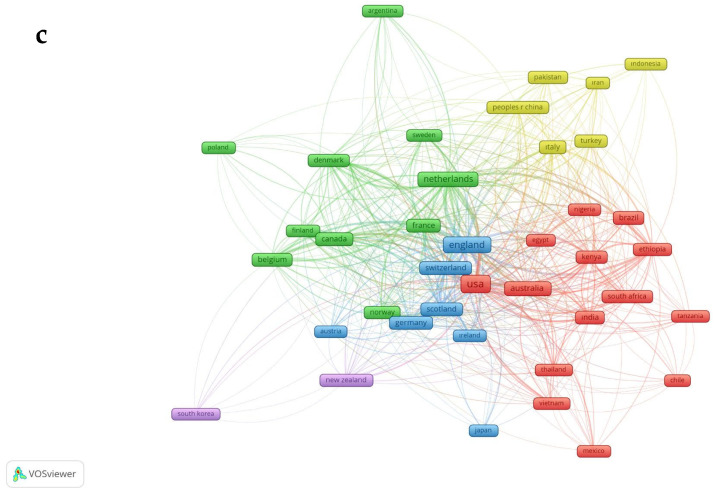
(**a**) Network visualization of citations among journals. (**b**) Network visualization of citations among organizations. (**c**) Network visualization of citations among countries.

For citation analysis among journals, the minimum number of documents of a source has been settled to 10 documents. Only 17 journals met the threshold, occurring in 5 clusters (Figure 12a). *Preventive Veterinary Medicine* was the journal with the highest TLS (299). It was in Cluster 2 and has strong citation links to the *Journal of Dairy Science* and *Transboundary and Emerging Diseases*. All of the journals were interconnected to at least two other journals.

#### 3.2.7. Citation Network: Organizations

In the citation analysis among organizations, only 38 institutions occurring in 4 clusters met the threshold, which is the minimum number of documents of an organization is 10 (Figure 12b). The cluster with the highest TLS included 8 institutions. Utrecht University was the institution with the highest TLS (320).

#### 3.2.8. Citation Network: Countries

For citation analysis among countries, the minimum number of documents for a country has been set to 10 documents. Only 40 countries met the threshold, occurring in 5 clusters (Figure 12c). The country citation network highlights England (TLS = 886), the USA (TLS = 846) and the Netherlands (TLS = 721) as the most influential countries in animal health economics with strong citation links across multiple nations. Countries such as Republic of Korea, Indonesia, and Pakistan exhibit limited citation links, indicating a need for enhanced international collaboration to improve research visibility and impact.

## 4. Discussion

Over the last 25 years, more research has been conducted in animal health economics due to a greater understanding of the economic impact of animal diseases, particularly around the mid-2010s, when numerous studies were conducted on the cost-effectiveness of disease management. This rise in research is consistent with the global demand for economic evaluations to explain how best to control livestock diseases such as foot-and-mouth disease (FMD) and avian influenza, as their control and management incur significant costs for governments and the livestock sector [1,3]. On the other hand, the rise in publication output is likely due to increased interest in the economic effects of zoonotic disease pandemics as well as the broader use of One Health approaches [6]. The general trend suggests that veterinary science and epidemiological economics are becoming critical interdisciplinary fields for animal health and economic sustainability [16].

The geographical distribution of authors demonstrates that high-income countries, particularly the USA, the UK, and the Netherlands, are dominant contributors to animal health economics. This research intensity is consistent with previous findings that well-established research institutions, access to funding, and strong academic networks increase scientific productivity [17]. The fact that middle-income countries such as India, Ethiopia, and South Africa are major contributors highlights the critical role of livestock in food security and rural livelihoods. The economic issues surrounding veterinary care are also becoming increasingly important in these regions [18]. However, the limited representation of authors from low-income countries reveals a significant gap in research contributions from areas where the economic burden of animal diseases is felt most [19]. These findings have broader implications that extend beyond descriptive patterns. The dominance of high-income countries highlights inequalities in knowledge production, limiting the applicability of results for regions most affected by animal health problems.

Economic impact and cost–benefit analyses are the dominant subjects of keyword and research analysis in animal health economics studies. Often cited studies like McInerney [2] and Rushton [1] emphasize that economic losses due to animal disease are not just in treatment expenses but also in productivity loss, trade restrictions and long-term market destabilization. The prominence of disease terms such as foot-and-mouth disease indicates that attention is being given to costly diseases affecting global livestock productivity. Furthermore, the utilization of terms such as vaccination, prevalence and risk factors indicates ongoing interest in prevention, supporting the demand for cost analyses and models to guide cost-effective disease control policy [11,13].

Through citation network analysis, we discover the foundational work that developed animal health economics research and recognize Hogeveen, H. and Rushton, J. as key influential figures. Hogeveen’s research in dairy economics emphasizes the financial advantages of disease management strategies within the livestock industry [12,20,21]. The research by Rushton J has been essential in developing new ways to assess the economic outcomes of disease control strategies. A minority of authors produce fewer publications yet achieve high citation numbers, which implies that their limited studies hold substantial influence within their field. The results highlight that research quality and methodological innovation matter more than just publication numbers.

European universities, such as Wageningen University and Utrecht University, lead research collaborations in their region, even while maintaining close connections with the University of Liverpool and the University of Edinburgh. The limited participation of developing nations in these networks highlights the need for increased involvement from developing regions in international research initiatives [22]. These stronger network connections would enable more exact local economic information on livestock disease management and improved knowledge exchange.

Using keyword co-occurrence analysis, the central research topics in animal health economics and their evolution over time were illuminated. When the keyword-occurrence analysis was examined, the most common keywords in the dataset were “economics”, “cost–benefit analysis”, “economic impact”, “foot-and-mouth disease”, and “vaccination”. The aggregation of zoonosis-related and economic burden-related terms suggests that scientists are particularly focused on the impact of animal health emergencies on global economies and public health systems. These results align with the growing recognition of the importance of integrating veterinary epidemiology with economic modelling in light of global pandemics, such as COVID-19 and influenza [5,23]. On the other hand, the fact that the most common animal species in the dataset is cattle suggests that future animal health economics studies should expand their analyses to include other species.

While there were areas that demonstrated strong collaborative work, the network mapping revealed significant fragmentation in the broader research scene. Of particular interest was the finding of multiple groups of authors in the emerging economies, with Indonesia and Pakistan among them. This finding implies that, despite increasing financial investment by these countries in research on animal health economics, their scientists may face difficulties in integrating with established international collaborations. González-Alcaide et al. [24] indicate that poor communication may be the reason for the low level of international cooperation among researchers from various nations, resulting in low international collaboration. Through open-access publishing models and global collaborative initiatives, these inequalities can be addressed and underrepresented areas integrated into the mainstream research network.

Another important finding from the coauthorship analysis was the evidence of robust multi-author collaborations, highlighted by high link numbers and total link strength rather than a simple temporal increase in author counts. This trend in animal health economics, more broadly, is represented in team-based work, where professionals from various fields, such as epidemiology, public health, and agricultural economics, collaborate on complex research. Interdisciplinary cooperation determines how challenging issues in animal husbandry, such as financial sustainability, food security, and disease outbreaks, are tackled. Wuchty et al. [25] mention that teamwork is increasingly replacing individual authors in scientific research and that multi-authored studies often have a greater impact. Therefore, further research in animal health economics is likely to continue drawing multidisciplinary, larger teams, thereby increasing the general influence and relevance of the subject.

Moreover, the co-occurrence network clearly shows how economic approaches have changed within veterinary science. Earlier studies concentrated mainly on direct economic costs, including treatment expenses and production losses associated with diseases such as mastitis and lameness [11,26,27,28] whereas recent research incorporates more comprehensive economic evaluation methods, including cost-effectiveness analysis, economic modelling, and risk assessment frameworks, particularly addressing vaccination, biosecurity, animal welfare, One Health, and public health aspects [29,30,31,32,33]. The change in approach illustrates the expansion of computing power and the availability of big data sets, which can allow researchers to conduct more complex estimations of economic effects. Future research directions may include the synthesis of big data analytics with machine learning and dynamic approaches in economics, which can enhance predictive power in economic analysis related to animal disease.

Such valuable information greatly facilitates understanding of this bibliometric analysis; however, we should acknowledge that it has its limitations. The research utilised the broad Web of Science Core Collection; however, it may have excluded some important publications indexed in Scopus, PubMed, or localised databases, potentially introducing a database bias. In addition, we only included studies published in English, which presents a language bias by excluding potentially relevant research in other languages. Nevertheless, our analysis incorporated studies from a wide range of countries and institutions worldwide, indicating that this bias did not substantially limit the global scope of the findings. Grey literature, such as reports, policy papers, and technical documents, was also not considered, which may restrict insights into applied aspects of animal health economics. The search strategy, although broad, may have overlooked some studies due to the use of different terms for the same concept. Addressing these limitations in future studies may enhance the depth and applicability of animal health economics.

## 5. Conclusions

This bibliometric analysis offers an insightful examination of the discipline of animal health economics between 2000 and 2024, with a specific emphasis on major trends in publication, key authors, collaborative research, and emerging thematic priorities. The study indicated that since 2014, there has been a steady increase in the rate of publication, primarily due to a growing awareness of the economic burden associated with animal diseases and the urgent need for effective control mechanisms. The USA, UK and the Netherlands are the top contributors, while surprisingly, developing countries that have sustained economic losses from livestock diseases mainly remain underrepresented in this area. Keyword analysis results reveal a continued focus on cost-effectiveness, economic burden, and policy evaluation while steadily moving toward preventive methodologies involving vaccination and prevalence. Network analysis identified highly influential authors and institutions, examining collaboration patterns that revealed European universities play a central role in influencing animal health economics.

There are still significant gaps in research efforts for future developments, despite some progress, particularly in the area of research from low-income countries, where the burden of livestock diseases is economically intensive. The increasing application of advanced economic modelling techniques offers a transition toward a more data-driven approach in animal health economics, with the potential to improve efficiency in disease control policy. However, the traditional cost–benefit analysis still dominates much of the field, limiting the scope of the research. Instead, more advanced modelling and interdisciplinary studies should be performed, and deeper connections between economic research and policy application need to be forged.

In addition, the expansion of bibliometric research to encompass broader sets of databases and various search approaches can potentially provide a more comprehensive view of the field. By overcoming these barriers, the study of animal health economics can significantly contribute to the establishment of evidence-based policy measures that advance global animal health, ensure agricultural sustainability, and foster economic resilience.

## Figures and Tables

**Table 1:** Top 10 authors with the highest h-index and their citation information.

Unit	All Citations	All Articles	h-Index
Hogeveen H	2667	40	22
Rushton J	1742	37	17
Häsler B	335	17	10
Rich KM	354	13	10
Stott AW	334	12	9
Halasa T	1025	9	9
Gunn GJ	317	11	9
Saatkamp HW	329	17	9
Torgerson PR	528	10	9
Zinsstag J	1778	11	9

**Table 2:** Top 10 most cited references.

References	No. of Citations
Rushton, J. (1999) [9]	50
McInerney, J. (1992) [10]	50
Knight-Jones, TJD. (2013) [3]	44
Seegers, H. (2003) [11]	42
Rushton, J. (2009) [1]	40
Halasa, T. (2007) [12]	38
Huijps, K. (2008) [13]	36
McInerney, J. (1996) [2]	35
Dijkhuizen, A. (1997) [14]	31
Bennett, R. (2003) [15]	31

## Data Availability

The original contributions presented in the study are included in the article/Supplementary Materials; further inquiries can be directed to the corresponding author.

## Supplementary Materials

The following supporting information can be downloaded at: https://www.mdpi.com/article/10.3390/ani15203006/s1, Table S1: Inclusion and Exclusion Criteria Applied in the Bibliometric Search; Figure S1: Flowchart of bibliometric search.
