# Supplementary material for: The Economics of Animal Health: A 25-Year Bibliometric Analysis
Source: Animals (Basel). 2025 Oct 16;15(20):3006. doi: 10.3390/ani15203006 (PMC12560921; doi:10.3390/ani15203006)
Supplement: Supplementary file 1 [file animals-15-03006-s001.zip › Table S1.pdf]

Table S1. Inclusion and Exclusion Criteria Applied in the Bibliometric Search

Criterion Type	Description
**Inclusion**	(i) Peer-reviewed journal articles and reviews; (ii) Published in English; (iii) Primary focus on the economic aspects of animal health (economic burden, cost-effectiveness, public health impact); (iv) Publications between 2000–2024
**Exclusion**	(i) Opinion pieces, editorials, non-research reports; (ii) Studies not published in English; (iii) Articles outside selected Web of Science categories (Veterinary Sciences, Infectious Diseases, Agriculture Dairy Animal Science, Parasitology, Economics, Zoology, Food Science Technology, Environmental Sciences, Fisheries, Agriculture Multidisciplinary, Agricultural Economics Policy, Environmental Studies, Tropical Medicine); (iv) Articles not related to animal health economics*

*This criteria was defined to ensure that the selected publications meaningfully contributed to the research landscape of animal health economics. Literature screening process was conducted focusing on publications addressing the economics of animal health. Inclusion criteria were established based on five key parameters: (1) the degree of economic orientation on animal health and diseases, (2) the application of economic concepts, (3) the discussion of control strategies within an economic context, (4) the presence of quantitative economic evaluation, and (5) the potential contribution to the field of animal health economics. Due to the interdisciplinary nature of the topic, the initial search returned a wide range of publications, including many categorized under human medicine-related WOS categories. To maintain focus on the animal health economics field, only publications within relevant categories were included. During the screening phase, a considerable number of articles related primarily to human health were also identified and excluded based on the five evaluation parameters. After applying these filters, a final dataset of 1070 publications were obtained for bibliometric analysis. The flowchart of the bibliometric search was given in Figure S1.
