# Supplementary figures and images for: The Economics of Animal Health: A 25-Year Bibliometric Analysis
Source: Animals (Basel). 2025 Oct 16;15(20):3006. doi: 10.3390/ani15203006 (PMC12560921; doi:10.3390/ani15203006)

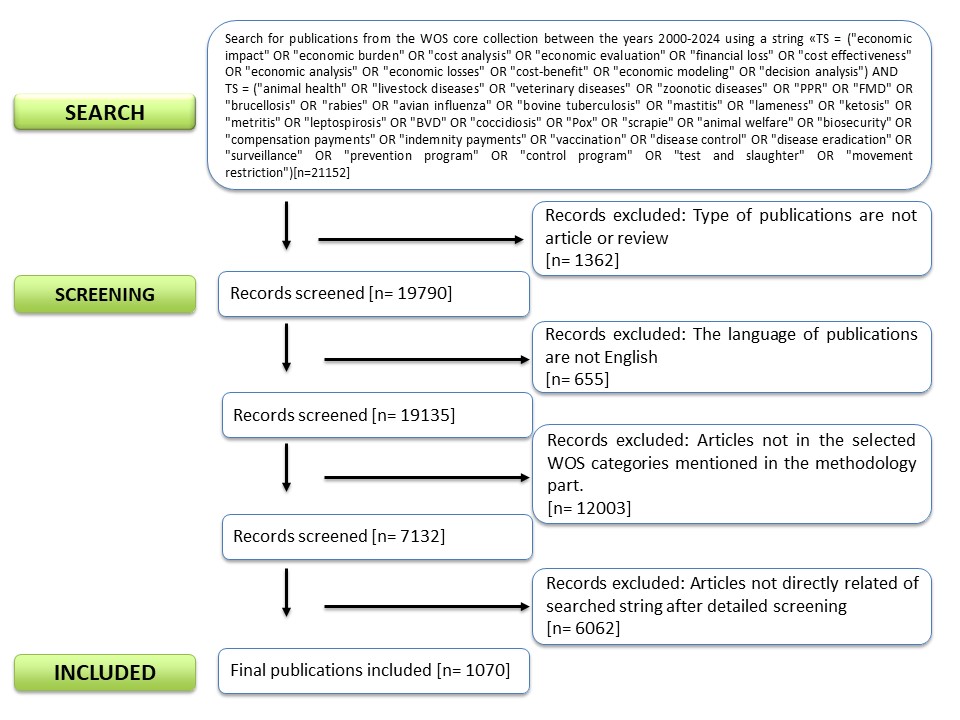

Supplement: Supplementary file 1 [file animals-15-03006-s001.zip › Figure S1.JPG]
